# Conditional associative learning examined in a paralyzed patient with amyotrophic lateral sclerosis using brain-computer interface technology

**DOI:** 10.1186/1744-9081-4-53

**Published:** 2008-11-24

**Authors:** IH Iversen, N Ghanayim, A Kübler, N Neumann, N Birbaumer, J Kaiser

**Affiliations:** 1 Department of Psychology, University of North Florida, Jacksonville, FL 32224, USA; 2 Institute of Medical Psychology and Behavioral Neurobiology, Eberhard-Karls-University, Tübingen, Germany; 3 Department of Psychology, Biological and Clinical Psychology, Julius-Maximilians-University, Würzburg, Germany; 4 Ospedale S. Camillo – IRCCS, Istituto di Ricovero e Cura a Carattere Scientifico, Venezia-Lido, Italy; 5 Institute of Medical Psychology, Goethe-University, Frankfurt am Main, Germany

## Abstract

**Background:**

Brain-computer interface methodology based on self-regulation of slow-cortical potentials (SCPs) of the EEG (electroencephalogram) was used to assess conditional associative learning in one severely paralyzed, late-stage ALS patient. After having been taught arbitrary stimulus relations, he was evaluated for formation of equivalence classes among the trained stimuli.

**Methods:**

A monitor presented visual information in two targets. The method of teaching was matching to sample. Three types of stimuli were presented: signs (A), colored disks (B), and geometrical shapes (C). The sample was one type, and the choice was between two stimuli from another type. The patient used his SCP to steer a cursor to one of the targets. A smiley was presented as a reward when he hit the correct target. The patient was taught A-B and B-C (sample – comparison) matching with three stimuli of each type. Tests for stimulus equivalence involved the untaught B-A, C-B, A-C, and C-A relations. An additional test was discrimination between all three stimuli of one equivalence class presented together versus three unrelated stimuli. The patient also had sessions with identity matching using the same stimuli.

**Results:**

The patient showed high accuracy, close to 100%, on identity matching and could therefore discriminate the stimuli and control the cursor correctly. Acquisition of A-B matching took 11 sessions (of 70 trials each) and had to be broken into simpler units before he could learn it. Acquisition of B-C matching took two sessions. The patient passed all equivalence class tests at 90% or higher.

**Conclusion:**

The patient may have had a deficit in acquisition of the first conditional association of signs and colored disks. In contrast, the patient showed clear evidence that A-B and B-C training had resulted in formation of equivalence classes. The brain-computer interface technology combined with the matching to sample method is a useful way to assess various cognitive abilities of severely paralyzed patients, who are without reliable motor control.

## Background

Forming relations among arbitrary stimuli is the hallmark of human learning. For example, we readily learn that a picture of an object can represent the object and that a printed word can represent both. Such relational learning among stimuli is formally studied as conditional associations or conditional relations, and the formation of equivalence classes among the stimuli is a highly relevant topic in both research and education (e.g., [1,2]). Given the ubiquity of equivalence class formation, recent studies have examined neural correlates of class formation [3,4]. Deficiencies in equivalence class formation have been examined in children with developmental disorders (e.g., [1]). Here we report on a method for assessment of equivalence class formation in one severely paralyzed patient with amyotrophic lateral sclerosis (ALS).

ALS can lead to motor disability so severe that the patient enters the "locked-in" syndrome. The patient can neither move nor speak and has therefore lost customary means of communication [5]. The general literature on ALS suggests that some patients have cognitive deficits and frontal lobe deterioration [6-8], but some studies find only few cognitive deficits and only in some tasks [9,10]. Assessing the cognitive abilities of patients at the late stage of paralysis is difficult because of their lack of reliable control of a motor response. Some late-stage ALS patients can learn to communicate reliably using only their EEG [11,12]. Patients can learn to control certain components of their EEG after biofeedback training and through a brain-computer interface can direct the movement of a cursor on a computer screen by regulating their EEG. Thus, the patient can use the EEG to make a voluntary response that requires no neuromuscular control but which can nonetheless be observed in the form of the visual feedback of the EEG change. The basic EEG-control task was used previously to teach ALS patients to compose words, letter by letter, using cursor movement to select characters of the alphabet [12-14]. The task has been described in detail elsewhere [12].

Using this EEG-control task, Iversen et al. [15] trained two severely paralyzed ALS patients to respond with high accuracy in a two-choice task so that they could answer questions relating to their cognitive skills. For example, a noun and a verb were presented, one in each choice target, and the patients were given the verbal instruction to steer the cursor to the noun on each trial. Similarly, other tasks assessed basic abilities such as: odd/even number discrimination, and discrimination of larger/smaller numbers. Performance was also assessed using a matching-to-sample paradigm [16], which was used to examine the ability to discriminate numbers, letters, colors, and to perform simple calculations. The two patients in Iversen et al. [15] performed above 90% correct on most tasks and thereby demonstrated that they understood the instructions, discriminated the stimuli, and could control the cursor by their EEG. Importantly, the method also detected that both patients had a possible deficit in tasks related to numerical computation, and one patient tested on delayed matching to sample showed inability to remember the sample stimulus at intervals longer than 5 s.

A task that is used for examination of acquisition of novel relations among stimuli is the so-called conditional-associative learning task, which tests the acquisition of arbitrary associations among visual stimuli (e.g., [17]). For example, Röttig et al. [10] presented a task to 15 non-bulbar ALS patients where the participants had to learn to associate each of six pictures with a meaningless pattern; the response was a distinct motor response of pointing to the correct design; none of the patients showed a deficit in this conditional-associative learning task. In general, performance on tasks that relate behavior to stimuli has been studied for a variety of brain injuries, including early work with aphasia patients (e.g., [18]).

**Figure 1:**
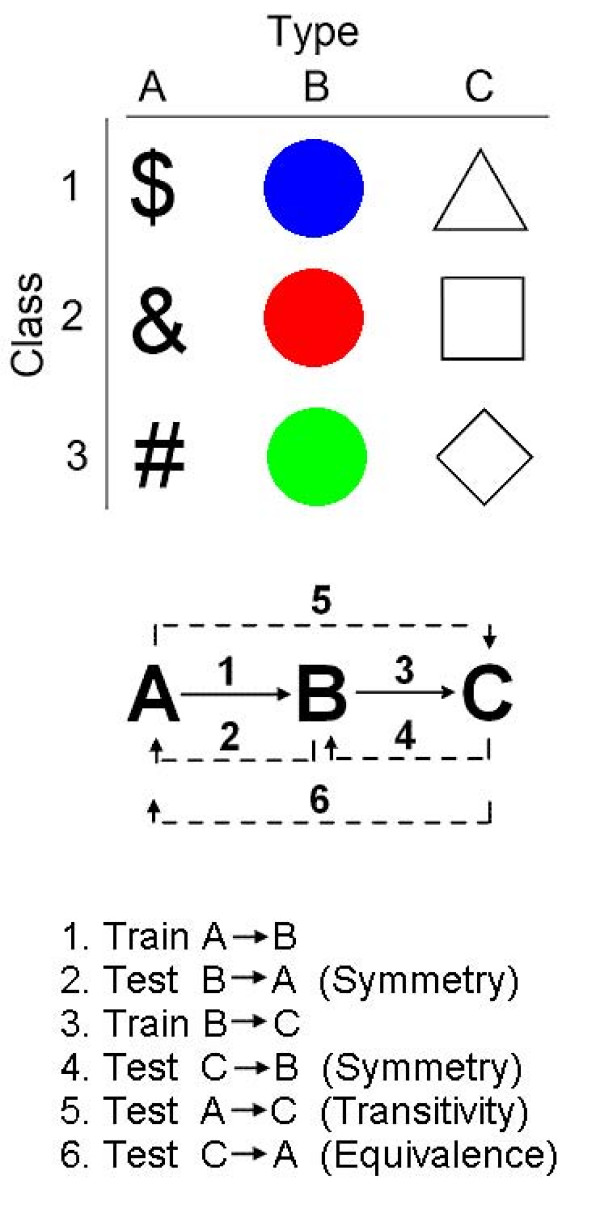
***Top*****: Schematic of the three types of stimuli used: signs, colored disks, and geometrical forms**. The objective of training was to form three classes of stimuli, with each class consisting of one sign, one color, and one geometrical form. *Middle*: Training related a stimulus from type A to a stimulus from type B, the A → B relations, and a stimulus from type B to a stimulus from type C, the B → C relations (solid arrows). All other relations among the stimuli were not trained (punctuated arrows). Instead, they were tested. *Bottom*: Sequence of relations trained and tested. Numbers refer to the arrows in the middle display.

The present research is a further development to study conditional relational learning in ALS patients. The experiment was designed to determine whether the patient could learn arbitrary conditional relations and whether the training formed classes of equivalent stimuli (e.g., [1,2]). Fig. 1 illustrates the logic of the experiment. The stimuli are of three types: signs, colored disks, and geometrical shapes. These stimuli are to be associated arbitrarily to form three classes with each class consisting of one sign, one colored disk, and one geometrical shape. Fig. 1, top, shows the stimulus types and the classes to be formed; for example, class 1 will consist of the three stimuli: a $ sign, a blue disk, and a white triangle. The training method is to show one stimulus from one type as a sample in the matching to sample method and have two stimuli as comparison or choice stimuli, the correct comparison is a stimulus from the same class as the sample, while the incorrect stimulus is from another class. Thus, for class 1, to teach the relation between type A and type B stimuli, or the A → B relation (sample → comparison), the sample on a given trial may be the $ sign and the two comparisons a blue disk and a red disk, with the blue disk being correct. On another trial, the sample may be the & sign with the two comparisons being one red and one blue disk, with the red disk being correct. Thus, whether red or blue is the correct choice is conditional on the sample being $ or &. Customarily with healthy human subjects, teaching the A → B and B → C relations makes the stimuli form classes of equivalent stimuli. Once the equivalence classes form, the subject can also correctly match all the untaught relations among the stimuli, and the stimuli show symmetrical, transitive, and equivalence relations [2]. The solid arrows in the middle part of Fig. 1 show the trained relations A → B and B → C. The punctuated arrows show the relations that the participant was not explicitly taught but should also have learned if the stimuli in each class became equivalent during training. Thus, to determine whether our patient had formed equivalence classes after training to do A → B and B → C relations, we tested the two symmetry relations B → A and C → B, the transitivity relation A → C, and the equivalence or reversed transitivity relation C → A. The specific aim of the study was to determine whether the ALS patient could use conditional association learning to form classes of arbitrarily related visual items and whether the class items had become equivalent.

## Method

### Patient

One male patient with advanced ALS participated in the study. Patient ER had sporadic ALS with disease onset at age 38 and was 44 at the time of the study. He had tetraplegia and respiratory weakness with incomprehensible speech. He had control over eye movements and rudimentary control of one foot. He was not artificially ventilated or fed. Patient ER was fluent in Turkish and could understand but not read German. The patient lived at home being cared for by family members and professional caregivers. Prior to the present study he had participated in EEG training for several months and had learned efficient control of cursor movement. Patient ER had been taught to use a program to communicate messages to his caregivers [19,20]. Using the same general method, he had also participated in a study that examined cognitive abilities with a matching to sample method similar to the one used in the present study [15]. In the present study, the patient was trained or tested once each or every other week in his home environment. Each training day usually lasted 2–3 h and consisted of several 5–10 min training sessions separated by 5–10 min breaks during which the patient relaxed and the trainer checked equipment. On a given training day, patient ER also had other training sessions unrelated to the present experiment.

#### Apparatus and recording

EEG was recorded from the vertex referenced to linked mastoids at a sampling rate of 256 Hz. The EEG signal was amplified using a conventional 16-channel amplifier (EEG 8, Contact Precision Instruments) with a low-pass filter of 40 Hz, and a high-pass filter of 0.01 Hz corresponding to a time constant of 16 s. Electrodes were 8 mm Ag/AgCl electrodes fixed with elefix cream at an impedance of less than 5 kOhm. Vertical eye movement was recorded with electrodes attached above and below one of the eyes. Slow cortical potentials (SCPs) were extracted from the on-line EEG signal and corrected for eye movement artifacts [21]. The criterion SCP amplitude changes were usually in the 30–50 μV range and were converted to visual feedback in the form of vertical movement of a small cursor on the monitor of a notebook computer (Toshiba Satellite 210CT), which was placed about 1 m from the patient's face [22].

#### Trial structure

**Figure 2:**
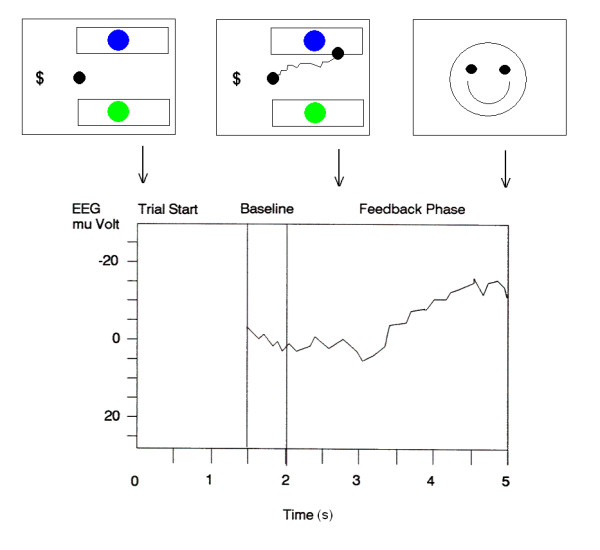
***Top*****: Schematic of the sequences of screens that the patient faced at trial start (left), during SCP-controlled cursor movement toward a target (middle, shows the path of cursor movement), and the final, brief reward screen of a "smiley" on correct trials**. *Bottom*: Schematic of the logic of SCP control. The average EEG during the last 0.5 s of the first 2 s following trial start was used as a baseline for subsequent EEG changes during the next 3 s. EEG was assessed relative to baseline during the last 0.3 s of the feedback phase to determine which target the cursor had reached.

Each training session (run) consisted of a series of 70 trials that each lasted 5 s. There were no inter-trial intervals. The patient faced the screen on the notebook computer that showed the sample at left, two targets at right, and a cursor between the targets (Fig. 2, top left). The onset of a trial was indicated by the presentation of stimulus material on the monitor and a 50 ms high-pitched tone. The cursor remained stationary on the monitor for 2 s (the preparatory phase). A within-trial baseline of SCP was recorded for the last 0.5 s of the 2-s preparatory phase (Fig. 2, bottom). This baseline served as an online immediate reference for SCP changes in the subsequent feedback phase, which lasted 3 s and was initiated by a 50 ms low-pitched tone [22]. The cursor began to move at a constant speed from left to right, 0.5 s into the 3-s feedback phase and moved for 2.5 s. The cursor's vertical movement was determined by the average SCP change relative to the 0.5-s baseline SCP value. During the feedback phase, SCP was updated every 62.5 ms and smoothed by a 500 ms moving average. If the patient produced a negative SCP change relative to the immediately preceding baseline the cursor moved up, and if the patient produced a positive SCP change relative to baseline the cursor moved down (Fig. 2, top middle frame). If the SCP remained the same as during the baseline, then the cursor did not move vertically. When the patient steered the cursor to the correct target, a "smiley" face appeared on the monitor as rewarding feedback during the last 0.2 s of a trial (Fig. 2, top right frame). The smiley did not appear if the cursor hit the incorrect target or if no selection was made or if a trial was rejected due to an eye-movement artifact or other artifacts such as from swallowing occurred [22]. A trial was repeated when the trial was rejected or when the cursor did not hit either target.

### Procedure

When the study began, the patient had prior experience in controlling the cursor on the computer screen by means of SCP changes in his EEG. Specifically, the patient had experience in controlling the cursor in matching to sample tasks using identical stimuli such as signs, colored disks, and geometrical shapes [15]. Thus, the patient had a prerequisite skill of identity matching with these stimuli at above 90% correct, with several sessions at 100% correct. Therefore, it was known that he understood the task, could control the cursor by his EEG, and that he could discriminate the stimuli that were to be used in the present experiment. Given this demonstration that these basic abilities were intact for this patient, possible performance decrements on the conditional relation task or in the formation of equivalence classes could be interpreted as indicative of possible deficits of the specific cognitive abilities related to formation of conditional relations.

The sample and the choices differed from trial to trial, and the location of the correct target varied randomly between the upper or lower position from trial to trial as well. Thus, the patient had to look at both the sample and the choice stimuli on each trial to solve the task correctly.

The stimuli were all within the size frame of 2 cm high and wide. Specifically, we used three types of stimuli with three elements of each type: three signs $, &, and #; three disks colored blue, red, and green; and three geometrical shapes triangle, square, and diamond (e.g., Fig 1). On each trial, one stimulus was presented as a sample on the left side of the monitor and two stimuli were presented as choices, with each choice stimulus centered in one choice box. The two choice stimuli were always of the same type (i.e., two signs, two colored disks, or two shapes), and the sample was always a different type. There was no requirement to make an observing response to the sample (which is a common requirement in research with conditional relations [2,16]); however, the patient obviously had to look at the sample to be able to solve the task. The patient had to learn which of the two choice stimuli was correct for a given sample stimulus. Because the symbolic stimulus relations were arbitrary (i.e., $ to Blue, & to Red, and # to Green), the task was different from the identity matching tasks that the patient was familiar with. The patient was instructed for both training and test sessions that he had to figure out by himself what was the correct stimulus.

The experiment was broken into 10 steps of training and testing. Table 1 shows the experimental condition, the procedure, and specific sessions for each step. The patient advanced from one step to the next when the accuracy was at or above 90% correct. For the first five training days, he was also maintained on identity matching with color stimuli, forms, signs, and on identifying a number in a string (for procedural details see [15]). On a given training day, such sessions were presented prior to or after the sessions of the present experiment. In all sessions, correct selections were followed by the smiley as feedback (e.g., Fig. 2). Ideally, selections on test trials consistent with equivalence class formation should not be followed by feedback [2]. During the period when the patient was available for the study, it was unfortunately not possible, for technical reasons, to prevent the smiley from appearing as feedback on test trials.

**Table 1:** Training and testing steps, brief description of procedure and number of sessions for each step.

Step	Condition	Procedure	Sessions
1	Training (A → B Relations)	A1 → B1, A2 → B2, A3 → B3	1, 2, 3, 4, 5
		A1 → B1, A2 → B2	6
		A1 → B1, A3 → B3	7, 8
		A2 → B2, A3 → B3	9, 10
		A1 → B1, A2 → B2, A3 → B3	11
2	Testing (B → A Symmetry)	B1 → A1, B2 → A2, B3 → A3 probes	12,13
3	Training (B → C Relations)	B1 → C1, B2 → C2, B3 → C3	14,15
4	Testing (C → B Symmetry)	C1 → B1, C2 → B2, C3 → B3 probes	16,17
5	Testing (A → C Transitivity)	A1 → C1, A2 → C2, A3 → C3 probes	18, 19, 20
		(Session 19: retraining of all A → B relations, session 11 repeated)	
6	Maintenance Training	Repetition of steps 2, 3, 4, and 5	21, 22, 23, 24
7	Maintenance Training	Relations within sessions:	25, 26
		A → B, B → A, B → C, C → B	
8	Testing (C → A Equivalence)	C1 → A1, C2 → A2, C3 → A3 probes	27, 28
9	Maintenance Training	Steps 2, 3, 4, 5 and 8 repeated	29 – 39
10	Whole Class Testing	Ax+Bx+Cx versus other combinations	40, 41, 42, 43

#### Step 1

Training A → B. The relation A → B was presented for the first five sessions using all three stimuli from each type. Thus, the presentations mixed within each session were A1 → B1 ($ to Blue), A2 → B2 (& to Red), and A3 → B3 (# to Green). Stimuli and their nomenclature are used as in Fig. 1. Because the patient did not learn the relations in these five sessions, the procedure was simplified to two relations each session until the patient achieved at least 90% correct. Thus, we presented A1 → B1 and A2 → B2 for one session, A1 → B1 and A3 → B3 for two sessions, and A2 → B2 and A3 → B3 for two sessions. Then all three relations were presented together again in session 11.

#### Step2

Symmetry testing of B → A. Once the A → B relations (all three relations) were learned, the patient was tested to see if he had also learned the reverse conditional discrimination. That is, the B stimuli were now presented as samples while the A stimuli were comparisons. During the two test sessions (12 and 13), the A → B stimuli from step 1 were presented as baseline trials while the B → A stimuli were presented in 12 probe trials (four for each of B1 → A1, B2 → A2, and B3 → A3). Probe trials were mixed with baseline trials in the symmetry testing sessions.

#### Step 3

Training B → C. The relation B → C was presented in two sessions using all three stimuli from each type. The presentations B1 → C1 (Blue to triangle), B2 → C2 (Red to square), and B3 → C3 (Green to diamond) were mixed within each session.

#### Step 4

Symmetry testing of C → B. Once the B → C relations in step 3 were learned, the C → B relations were tested by presenting the C stimuli as samples and the B stimuli as comparisons in 12 probe trials while the B → C relations from step 3 were presented as baseline trials.

#### Step 5

Transitivity testing of A → C. After the symmetry tests, the transitive relation A → C was tested with an A stimulus as sample and two C stimuli as comparisons. The 12 A → C probe trials were mixed with B → C relations as baseline trials. A procedural error occurred prior to the first transitivity test. The trainer had inadvertently missed to run one session from the protocol with the method of step 1 to reacquaint the patient with the A stimuli as samples, which had been absent during steps 3 and 4. After the first test session of transitivity, the patient therefore had one session with A → B trials; that is, the method from session 11 was repeated in session 19. Then session 20 presented one more transitivity test.

#### Step 6

Maintenance training. For four sessions, the training was simply a repetition of sessions from steps 2, 3, 4, and 5.

#### Step 7

Maintenance training with mixed relations within sessions. As a preparation for the next equivalence test, trials in a session were mixtures of the A → B, B → A, B → C, and C → B relations.

#### Step 8

Equivalence testing of C → A. The reversed transitivity relation C → A was tested by having the C stimuli serve as samples for choices between A stimuli in 12 probe trials. The baseline trials were as in step 7 except that the C → B relations were omitted.

#### Step 9

Maintenance training. The procedures from steps 2, 3, 4, 5 and 8 were repeated for 11 sessions.

#### Step 10

**Figure 3:**
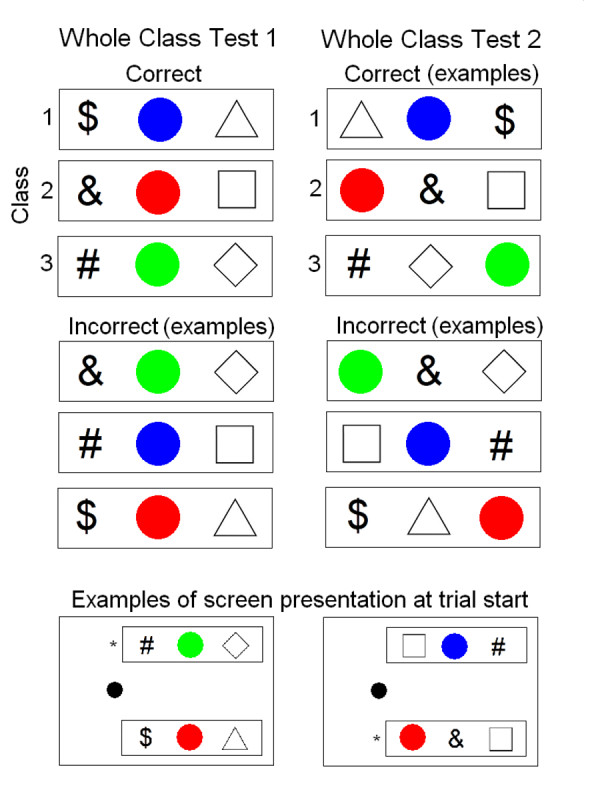
**Display of the stimulus presentation method for the Whole Class Tests**. All three stimuli in a class were presented together at the same time in one choice box while the other choice box presented a mixture of stimuli from the two remaining classes or stimuli across all three classes. No sample stimulus indicated which choice box was correct. In Test 1, the stimuli were presented together in a box in the order of sign, colored disk, and geometrical form, from left to right. In Test 2, the stimuli appeared together in random order. Notice that the correct stimuli can only be presented in one order in Test 1 whereas there are many permutations of presenting the incorrect stimuli. Similarly, for Test 2, there are many permutations for presenting both the correct and incorrect stimuli, therefore only examples are shown. The bottom two frames show examples of what the screen looked like. The asterisks indicate the correct boxes; this sign was not shown on the monitor.

Whole class testing. The formation of equivalence classes was tested in an additional, novel way by presenting the three stimuli from one class together as a group in one choice box and three stimuli mixed from the two other classes or from all three classes in the other choice box. For example, $, Blue, and Triangle "go together" as a group of stimuli because they form one equivalence class whereas the stimuli #, Blue, and Square do not "go together" as a group because they do not form an equivalence class. In this test there was no sample presented on the left side of the monitor. The patient had to move the cursor to the box that showed the stimulus group that formed a class through training; this group was considered correct while the other group was considered incorrect. Fig. 3 shows examples of the stimulus groups in the choice boxes. For whole class test 1, both correct and incorrect stimulus groups presented the stimuli in the order of type of A, B, and C from left to right. For whole class test 2, stimuli were presented in mixed order for both correct and incorrect stimulus groups. Notice that there are many permutations of presenting three stimuli in mixed order and that only some of them are shown in Fig. 3. The bottom display in Fig. 3 shows examples of how the screen was presented at trial start for each type of test; notice the absence of a sample stimulus. Thus, this whole-class test is not a conditional discrimination but a simple two-choice test for discrimination of whole classes of stimuli. All trials in a session were of this type. Two sessions were given for test 1 and two sessions were given for test 2.

Table 2 shows an overview of how the sessions were distributed over the 10 days that the trainer visited the patient at home. Because the patient took part in other experiments on the same days, the trainer was not always able to follow the prescribed protocol, which resulted in some days having only one or two sessions.

**Table 2:** Distribution of sessions over the 10 days that the patient was trained and tested.

Day	Session
1	1, 2
2	3 – 11
3	12
4	13 – 20
5	21 – 28
6	29 – 35
7	36, 37
8	38
9	39 – 41
10	42, 43

## Results

During the first five training days where the patient had identity matching sessions using the same stimuli as in the conditional relation procedures, the patient maintained a high level of accuracy ranging from 88.6% to 100%, with an average of 96.3% (N = 19 sessions); six sessions were at 100%.

**Figure 4:**
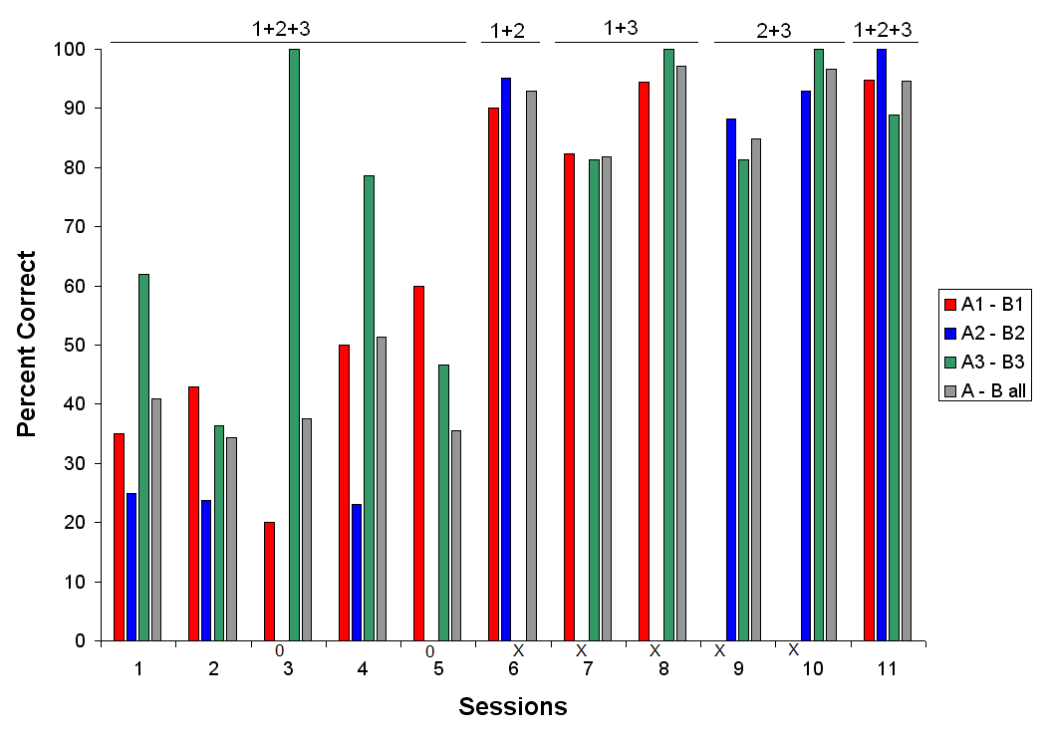
**Percent correct for each of the A → B relations (1 through 3) and all three relations together**. Numbers above the graph indicate which relations were presented in mixed order within a session. Along the X-axis, 0's indicate a score of zero, and x's indicate that a given relation was not presented in a session.

The percent correct for each of the 11 sessions of teaching the A → B relations is presented in Fig. 4. Data are shown for each of the three relations (A1 → B1, A2 → B2, and A3 → B3) and for all relations together. During the first five sessions with all relations presented in mixed order within sessions, the patient did not learn the relations and even was at 0% correct for the A2 → B2 trials in sessions 3 and 5. Therefore, the procedure was simplified to present only two of the relations within each session until the patient learned the relations. The percent correct immediately increased. When all three relations were again presented in mixed order in session 11, the patient scored at or above 90% correct on all three relations and had therefore learned the arbitrary conditional association between the type A stimuli (signs) and the type B stimuli (colored disks).

**Figure 5:**
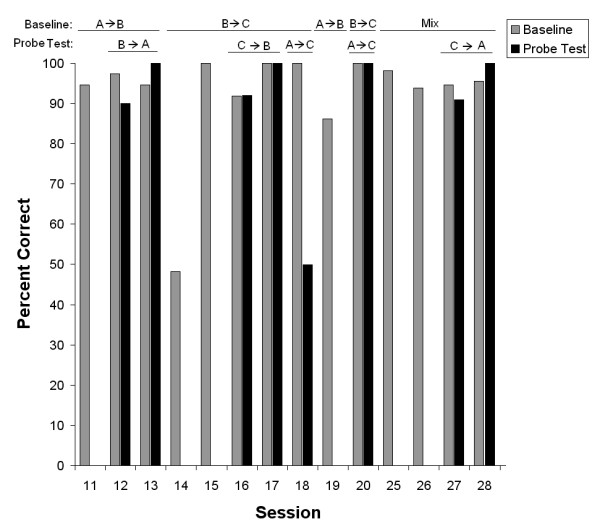
**Percent correct for all trained and tested relations through steps 2 to 8**. Relations above the graph indicate baseline relations and probe test relations. All indications are from Sample → Comparisons. Data are shown for both baseline trials and probe test trials; some sessions did not have probe test trials. Data from step 6, sessions 21 through 24, where steps 2, 3, 4 and 5 were repeated, are not shown to save space.

Fig. 5 shows percent correct for baseline and test trials for the symmetry, transitivity, and equivalence tests in steps 2 through 8. On the B → A symmetry tests, patient ER maintained a high accuracy on baseline trials and was 90% and 100% correct on probe trials showing clear evidence that the learned A → B relations were symmetrical without explicit training.

After the B → A symmetry test, the patient was taught the B → C relations (all three at the same time) in sessions 14 and 15 and learned all three in those two sessions in contrast to the slow learning of the A → B relations in sessions 1 though 11. On the C → B symmetry probe trials, patient ER scored 92% and 98% correct indicating that the taught B → C relations were symmetric. The A → C test for transitivity was given prematurely on session 18 by mistake (see procedure), and the patient scored at chance level on probe trials while maintaining the baseline at close to 100% correct. Because the patient had not been exposed to the A → B relations for some sessions, this relation was reintroduced in session 19. A new A → C transitivity test in the following session 20 showed nearly 100% correct on both baseline and probe trials indicating that the taught A → B and B → C relations were transitive.

To prepare the patient for the C → A equivalence test, two sessions presented all the necessary trial types (see Table 1) in mixed order for sessions 25 and 26, and the overall accuracy was above 90%. On the two C → A equivalence tests, the patient scored 90% and 100% correct, respectively, indicating that the taught A → B and B → C relations were equivalent; the baseline accuracy was close to 95% in both sessions.

After the equivalence test, steps 2, 3, 4, 5 and 8 were repeated as step 9 over 11 sessions (sessions 29 – 39) to maintain the overall performance. Accuracy on baseline and probe trials was above 90% on each of these sessions, and data are not shown to save space.

The last test involved a choice between groups of stimuli; one group showed three equivalence class-related stimuli together while the other group showed three stimuli that did not constitute an equivalence class. All trials in these test sessions were of this type (two test types were presented, see procedure). Each test was presented twice. For the first two sessions, the accuracy was 94.4% and 98.3% for Test 1 and Test 2, respectively. For the next two sessions, the percent correct was 98.3 for both Test 1 and Test 2. That the overall correct selection was very close to 100% on all four sessions indicates that the patient easily identified and selected the group of stimuli that formed an equivalence class.

**Figure 6:**
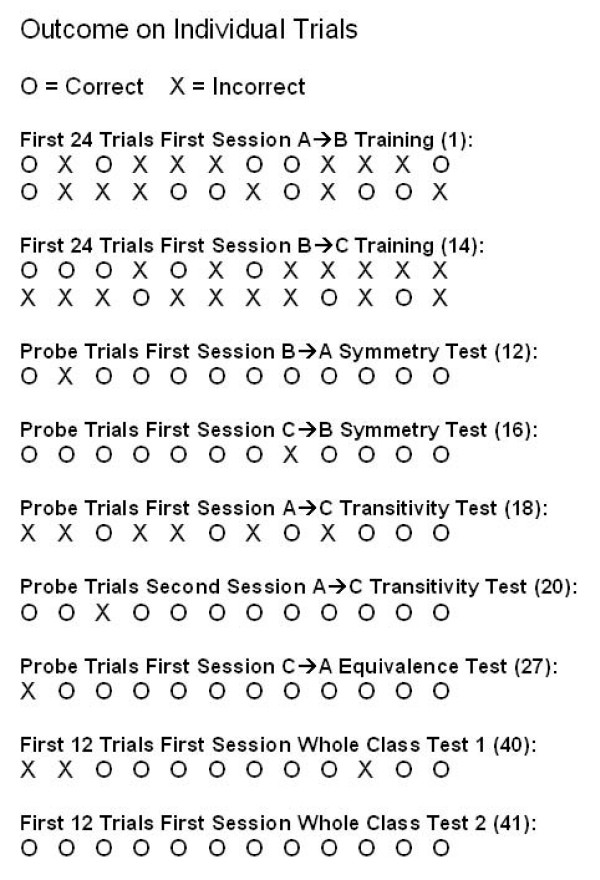
**To assess possible learning within sessions, performance on individual trials (correct or incorrect) is shown for selected sessions**. Session number is indicated in parenthesis for each data set. For the first teaching sessions with A → B and B → C relations (sessions 1 and 14, respectively), the data are shown for the first 24 trials of each session. For the test sessions with B → A, C → B, A → C, and C → A relations (sessions 12, 16, 18, 20, and 27), the data are shown for all probe trials (12 per session). For the whole class tests, data are shown for the first 12 trials of the first session of each test (sessions 40 and 41).

Because the patient received a smiley as positive feedback on all correct trials through the experiment, a natural question is whether the overall correct performance on probe test trials reflected learning during prior training sessions or reflected quick learning during the probe trials. To answer this question, the data were analyzed at the level of individual probe trials for the first session of each test type. Fig. 6 shows correct or incorrect selection on each trial of probe testing (12 trials each test session) and in comparison also for the first 24 trials of the first session of A → B training and the first session of B → C training, where the stimulus relations were new to the patient. If the probed relations had to be learned during testing, one would expect probe trial performance to resemble performance during sessions with learning of new relations. The A → B and B → C relations were learned slowly over several sessions (11 sessions for the A → B relation and 2 sessions for the B → C relations, Figs. 4 and 5), and correct selections in the beginning of the first sessions of these relations did not lead to quick learning (Fig. 6). In contrast, during probe trials in test sessions, the patient made very few if any mistakes from the beginning of probe trials, indicating that the relations the probes trial tested for were not learned during testing. Only during the first A → C probe test did the patient perform poorly, as already described above, and there was no indication of quick learning after one or two correct trials (Fig. 6). Thus, the overall data suggest that the patient did not learn the test relations from feedback during the first few probe trials at the beginning of a test session. Instead, the performance on probe test trials indicated that the patient selected the correct stimulus during probe testing based on learning during prior training sessions.

## Discussion

The patient reliably matched correctly when the stimuli were used in identity matching indicating that he understood the task, could discriminate the stimuli, and could control the cursor with the SCP of his EEG. When instructed to learn on his own the arbitrary conditional associations between the stimuli from type A and B, the A → B relations, he showed no learning after five sessions, and the task had to be broken into simpler steps of only two different associations per session before he could learn it. The next B → C relations were learned much faster in two sessions with all three associations presented within each session.

When tested for symmetry, transitivity, and equivalence, the patient scored at or above 90% indicating that A → B and B → C training had formed equivalence classes of the trained stimuli. However, on the first transitivity test (A → C) he scored at chance level. Because of a logistic mistake, the patient was not given a reminder session of the A → B relations (after B → C training and C → B symmetry testing) before the A → C transitivity test, and that may have resulted in the low score because his transitivity test was at high accuracy after the reminder session. The patient was also tested for discrimination of whole classes in a novel test. He could easily select the group of stimuli that formed a class over a group of stimuli that did not form a class. Whole class tests may resemble a few cases of reported sorting or categorization of equivalence class stimuli (e.g., [23,24]). Such tests may be useful supplements to customary equivalence testing.

The smiley was presented after a correct selection for all session types, including test probes. Customarily in testing for equivalence classes, subjects are not informed during testing whether they are correct or not [2]. This is done to avoid teaching the new relations during testing. Ideally that should also have been the case in the present experiment. However, at the time when the experiment was carried out it was impractical to modify the existing, hardwired feedback procedure of presenting a smiley after a correct selection. Evidence in our data suggests that the patient most likely did not learn the tested relations during testing. First, the acquisition of the A → B relations took 11 sessions and the acquisition of the B → C relations took two sessions. This indicates that for this patient, learning new relations was not instantaneous after just a few correct trials. Second, an analysis of performance on individual probe trials suggested that the patient selected correctly from the beginning of probe test sessions, which indicates that the relations were acquired as a result of learning in prior training sessions. Third, on the first test session for transitivity (A → C relations), the patient did poorly on probe trials throughout the session indicating that he did not learn quickly from just a few correct trials. Thus, collectively, these pieces of evidence suggest that in the present experiment, the equivalence classes did form during training and not during testing.

The diagnostic instrument developed here is for use with severely paralyzed persons, who cannot be adequately examined for possible cognitive deficits with traditional neuropsychological instruments that require a reliable motor skill of some form. The tasks presented here required pretraining to learn to use the SCP of the EEG to control the brain-computer interface. Control participants were not used. Instead, a within-subject design was used to compare the patient to himself as a control [25]. Thus, when the patient was excellent on one task (e.g., identity matching) but performed poorly on a second similar task (e.g., conditional relation of A → B) on the same training day, using the same stimuli, the inference can be drawn that the patient did not lack the skill to discriminate the stimuli or the skill to control the cursor; instead he lacked some ability to process information for the second task. Thus, with this single-subject design, the tentative conclusion could be reached that the late-stage ALS patient we tested may have had some selected cognitive deficits, specifically in terms of ability to acquire simple conditional associate learning relations.

The same patient was tested with delayed identity matching using geometrical forms as stimuli [15]. Without delay between sample and comparisons, the patient was close to 100% correct but with a delay between sample of comparison of 5 s or longer, the patient scored at chance level indicating a possible severe deficit in short-term memory. It is conceivable that the deficit in acquisition of the A → B relation in the present experiment, which the patient did not learn at all within five sessions of exposure to the task, reflects the same deficit he showed in the short-term memory test. To learn arbitrary associations between stimuli, the participant has to remember what happened on previous trials within the past few minutes in a given session to learn from the feedback. The literature indicates that healthy subjects can acquire very complex conditional associations within a time span much shorter than it took the patient in the present experiment (e.g., [23]). Once a relation is acquired, and once relations form equivalence classes during training, then responding correctly during equivalence testing may, loosely speaking, be a matter of long-term memory [26,27], for which the patient showed no deficit.

Studies of equivalence class formation in brain-injured patients are relevant for an understanding of brain mechanisms and also for the development of teaching methods for patients who may have lost skills due to injury or disease. Thus, conditional-relations training can be used to teach more relations than are presented in training. In the present experiment only two relations were explicitly taught, the A → B and the B → C relations, while the remaining relations actually emerged through the training [2]. Thus, this type of training can be used to teach new skills to patients with brain injury. For example, similar conditional-relation training was used to teach emotion naming [28] or face naming [24] to adults who had lost such skills due to brain injuries.

One limitation of the present study is that the ability to control the SCP of the EEG is a prerequisite skill for responding correctly in the two-choice task. Such training to control the SCP may take weeks, and some ALS patients do not learn the skill [12,22]. An alternative approach would be to use a different type of BCI which operates on the basis of event-related potentials (P300) and allows for presentation of at least four choices [29-31]. This method does not require lengthy training.

## Conclusion

The main impetus of the present research was to develop a method that could assess cognitive function in late-stage paralyzed ALS patients with no remaining reliable motor control. With only one patient, the present results should be considered preliminary. Thus, while there may be cognitive deficits associated with ALS, such as slowness in acquiring new conditional associations, it should be reemphasized that the patient did form equivalence classes of the stimuli, as healthy human subjects ordinarily do. Thus, even late-stage, locked-in patients, who may appear completely separated from their world in terms of communication, actually still may have considerable cognitive abilities and can be enabled to express their thoughts and wishes by means of brain-computer interfaces [31-33].

## Abbreviations

ALS: amyotrophic lateral sclerosis; EEG: electroencephalogram; SCP: slow-cortical potential.

## Competing interests

The authors declare that they have no competing interests.

## Authors' contributions

IHI, AK, NB and JK designed the study. NG, NN and JK executed the study. IHI analyzed the data, prepared figures, and developed the manuscript. All authors were involved in the editing and approval of the final manuscript.
